# Effect of Defects on Progressive Failure Behavior of Plain Weave Textile Composites

**DOI:** 10.3390/ma14164363

**Published:** 2021-08-04

**Authors:** Kyeongsik Woo, Jae Hyuk Lim, Cheolheui Han

**Affiliations:** 1 School of Civil Engineering, Chungbuk National University, Cheongju 28644, Korea; 2 Department of Mechanical Engineering, Jeonbuk National University, Jeonju-si 54896, Korea; jaehyuklim@jbnu.ac.kr; 3 Department of Aeronautical and Mechanical Design Engineering, Korea National University of Transportation, Chungju-si 27496, Korea; chhan@ut.ac.kr

**Keywords:** plain weave textile composites, voids, interface separation, progressive failure, cohesive zone modeling

## Abstract

Various types of internal defects occur during manufacturing and handling of composite materials. It is practically impossible to manufacture composite structures without defects, making it crucial to understand the effect of defects on their failure behavior to maintain structural safety. In this work, the effect of pre-defects on the failure behavior of plain weave textile composites was studied. Unit cell configurations with symmetric, in-phase, and shifted fiber tow arrangements were considered. Inter-laced warp and fill tows and matrix pockets of plain weave unit cells were modeled in three-dimensional finite elements, and cohesive elements were inserted between all bulk elements to account for the fracture modes of the fiber and matrix direction failure of warp and fill tows, matrix pocket failure, and interface failure. Unit cell models containing pre-defects of voids, tow-matrix pocket separation, warp-fill tow separation, and cracks in the warp and fill tows were analyzed, and their effects on progressive failure behavior were investigated in terms of the interaction between fiber tow arrangements and defects. Results indicated that initial failure occurred in matrix-direction failure mode in fill tows, whereas fiber tow-matrix pocket separation was the major failure mode under uniaxial tensile load. Furthermore, failure behavior was found to be highly dependent on the fiber tow arrangement pattern and the location of pre-defects.

## 1. Introduction

Textile composite materials have various fiber tow architectures depending on the weaving style. Generally, they are fabricated by crisscrossing fiber bundles in various directions to weave preforms and then by filling the matrix material between the empty spaces. The multidirectional arrangement of fiber bundles affords textile composites several advantages over conventional metal materials and unidirectional composite materials, including excellent formability into complex shapes, balanced properties in all directions, and excellent resistance to out-of-plane impact [1,2,3]. Due to these advantages, textile composite materials are used in advanced aerospace structures, as well as in various fields such as lightweight automobiles, special ship structures, construction materials, and medical and sports goods [4,5].

Extensive studies involving textile composites have been conducted since the early 1980s, including Ishikawa and Chou’s work on the property characterization of textile composites [6,7]. Such studies were initially focused on predicting the equivalent properties [8,9] and failure strengths [10,11], but subsequently the progressive failure of textile composites was studied numerically with the development of numerical fracture mechanics methods [12,13,14].

Composites are known to be defective in advance during the molding and production process [15,16,17,18], and textile composite materials having complex fiber tow structures compared to unidirectional composite materials have more diverse types of defects. Fabrication defects in textile composite materials can be divided into fiber defects (fiber fracture), matrix defects (void and incomplete curing), and surface separation defects (fiber and matrix separation and layer separation) depending on the location of the constituents [19,20]. Void defects are caused by the incomplete filling of matrix materials during the curing process, accounting for the highest frequency of defects in composite materials. Moreover, fiber tows can be damaged during handling, fiber misalignment can occur during lay-up, and defects can develop during processing, such as drilling and joining [21,22].

Defects in composite materials are known to have a large impact on material properties and failure behavior, but the manufacturing of composite structures without defects can increase production costs significantly. High-tech aerospace structures, which generally prioritize safety and mission accomplishment, do not permit void fractions of more than 1% by means of high-level molding and production and quality control techniques, which significantly increase production costs [23,24,25]. For example, it has been reported that the rejection percentage of drilled composite parts within 1% of voids is 60% in aircraft structures [23]. Conversely, industries that have relatively less severe weight requirements and are more sensitive to manufacturing costs such as auto parts, ship structures, and wind power rotor blades allow certain defects for economic reasons [26]. In such cases, allowing defects of approximately 5% has been shown to result in a 10% reduction in structural performance but a 40% reduction in fabrication costs [23].

It is practically impossible to manufacture composite structures without defects. Therefore, to design safe and economical composite structures, the effect of defects on structural integrity must be understood. For unidirectional composites, research has been conducted on the detection of fabrication defects and their effect on stiffness and strength reduction [27,28,29,30]. However, in the case of textile composites, defect detection studies have appeared only recently [31,32], and a limited number of studies have been performed on the effect of defects on stiffness and strength degradation [33,34]. More research is needed to better understand how existing defects can reduce the performance of textile composite structures.

In this study, the effect of manufacturing defects on failure behavior was studied numerically for plain weave textile composites. Interlaced warp and fill tows and matrix pocket materials of plain weave unit cells were discretely modeled using three-dimensional (3D) finite elements, and failure behavior was accounted for by using cohesive zone modeling (CZM). To fully consider the fracture modes of the fiber and matrix direction failure of warp and fill tows, matrix pocket failure, and interface failure, cohesive elements were inserted between all bulk elements, and selective activation of the cohesive element method [35,36] was employed to expedite the computation. In the analysis, unit cells of symmetric and in-phase fiber tow arrangements in the thickness direction were considered, as well as two-ply shifted unit cells. Unit cell models containing pre-defects of voids, tow-matrix pocket separation, warp-fill tow separation, and cracks in warp and fill tows were analyzed, and their effects on progressive failure behavior were investigated in terms of the interaction between fiber tow arrangement and defects.

## 2. Analysis

### 2.1. Configuration

**Figure 1:**
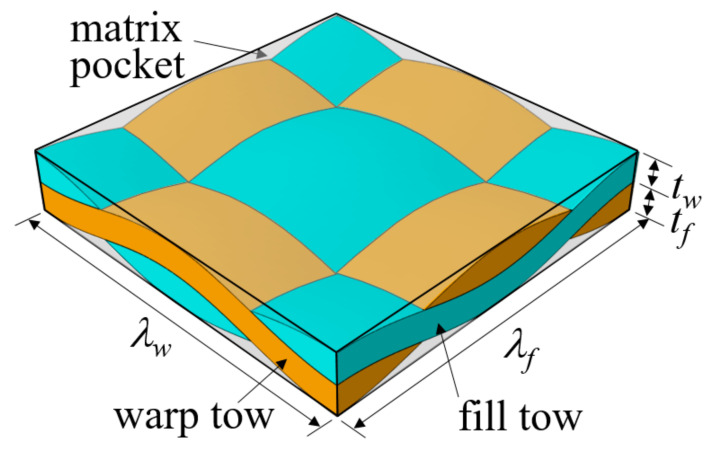
Fiber tow structure of plain weave textile composites.

Plain weave textile composites are constructed by first interlacing warp and fill tows, followed by impregnation with resin. Figure 1 shows a typical fiber tow structure of plain weave composites. Warp and fill tows are interlaced with each other, and matrix material is impregnated between the fibers in the tow region. The empty region is also filled with a matrix, resulting in a pure matrix pocket. The fiber tows are assumed to have a lenticular cross section and a sinusoidal tow path. In this case, the microstructural geometry of plain weave composites can be defined by the wavelengths ($\lambda_{w}$,$\;\lambda_{f}$) and heights ($t_{w},t_{f}$) of the warp and fill tows. The constituent materials considered in this study were AS4 fibers and 3501-6 epoxy matrix. Table 1 summarizes the microstructural dimensions [13], and Table 2 and Table 3 summarize the elastic and cohesive properties of the fiber tow and matrix [37,38]. The waviness ratio of the fiber tows is defined as λ/t where $t=t_{w}+t_{f}$, is 0.167.

### 2.2. Unit Cell Analysis

Unit cell analysis is often performed to approximate the behaviors of large complex structures, which are either impossible or impractical to simulate and require huge computational resources. A unit cell is defined as a minimum periodically repeating block of structures. The periodicity of a unit cell includes not only its geometry but also the solution behavior, which is obtained by applying periodic boundary conditions (PBCs) to the unit cell boundary [39,40].

**Figure 2:**
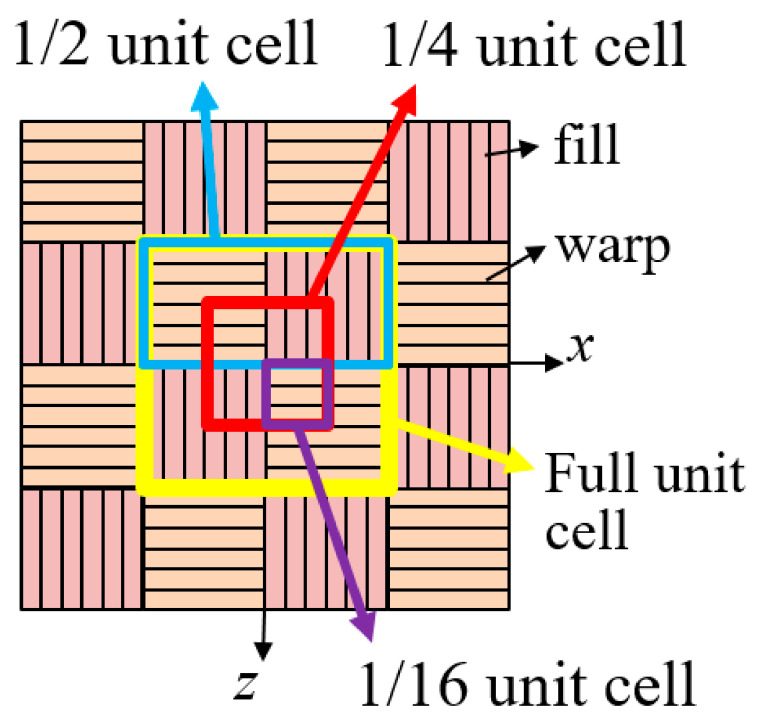
Full and partial unit cells of plain weave textile composites.

**Figure 3:**
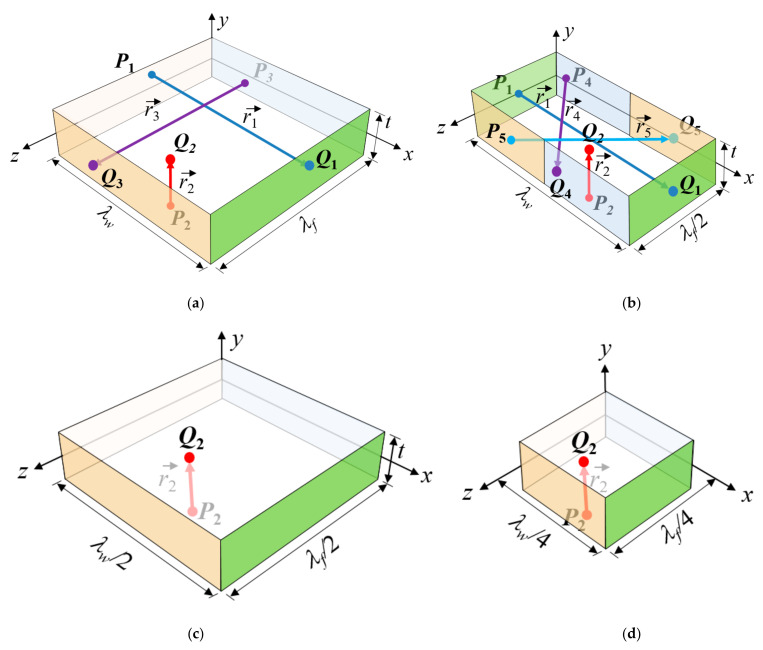
Periodicity vectors of full and partial unit cells: (**a**) full unit cell; (**b**) 1/2 unit cell; (**c**) 1/4 unit cell; and (**d**) 1/16 unit cell.

Plain weave composites exhibit periodicity in the fiber tow structure. Figure 2 shows the full and partial unit cells of plain weave composites. For a full unit cell, as shown in Figure 3a, the geometric periodicity is represented by the following equation:

(1) $${\vec{x}}_{\alpha }^{P}+{\vec{r}}_{\alpha }={\vec{x}}_{\alpha }^{Q}$$

where ${\vec{x}}_{\alpha }^{}$ and ${\vec{r}}_{\alpha }$ are the position vector and periodicity vector, respectively. The superscripts *P* and *Q* denote two nodes located in the matching boundary surfaces, and the subscript *α* is the surface pair number. For a plain weave unit cell, the sizes in the *x*-, *y*-, and *z*-directions are defined by the wavelengths of the warp and fill tows ($\lambda_{w}$,$\;\lambda_{f}$) and the specimen thickness (t), and the periodicity vectors are written as:

(2) $${\vec{r}}_{1}=\lambda_{w}{\vec{e}}_{1}^{},\;{\vec{r}}_{2}=t{\vec{e}}_{2}^{},\;{\vec{r}}_{3}=\lambda_{f}{\vec{e}}_{3}^{}$$

Then, the PBCs can be defined by the following equation:

(3) $${\vec{u}}_{\alpha }^{P}+\hat{\varepsilon }\left({\vec{r}}_{\alpha }\right)={\vec{u}}_{\alpha }^{Q}$$

Here, $\vec{u}$ is the displacement vector and $\hat{\varepsilon }$ is the meso-scale nominal strain tensor of the unit cell.

The PBCs to simulate tensile test in the x-direction can then be derived as:

(4a) $$u_{1}^{P}+{\hat{\varepsilon }}_{11}\lambda_{w}=u_{1}^{Q},\;v_{1}^{P}=v_{1}^{Q},\;w_{1}^{P}=w_{1}^{Q}$$

(4b) $$u_{2}^{P}=u_{2}^{Q},\;v_{2}^{P}+{\bar{\varepsilon }}_{22}t=v_{2}^{Q},\;w_{2}^{P}=w_{2}^{Q}$$

(4c) $$u_{3}^{P}=u_{3}^{Q},\;v_{3}^{P}=v_{3}^{Q},\;w_{3}^{P}+{\bar{\varepsilon }}_{33}\lambda_{f}=w_{3}^{Q}$$

In the above, the chevron and over-bar accents are used to indicate the specified value and unknowns determined during the analysis, respectively. The PBCs for the other loading conditions can be similarly derived.

For plain weave composites, it is possible to use a 1/2 unit cell with a size of ($\lambda_{w}$,t,$\lambda_{f}/2$) as shown in Figure 3b with modified PBCs, instead of the full unit cell. (Reducing the model size can be an important issue because progressive failure analyses often require extensive computational time.) In this case, the periodicity vectors ${\vec{r}}_{1}$ and ${\vec{r}}_{2}$ in the warp tow and thickness directions, respectively, are the same as those in Equation (2), and thus are the PBCs in (4a) and (4b). However, the outer surfaces normal to the *z*-direction (the fill tow direction) are divided into two groups that are connected by the following periodicity vectors:

(5a) $${\vec{r}}_{4}=\left(\lambda_{w}{\vec{e}}_{1}+\lambda_{f}{\vec{e}}_{3}\right)/2$$

(5b) $${\vec{r}}_{5}=\left(\lambda_{w}{\vec{e}}_{1}-\lambda_{f}{\vec{e}}_{3}\right)/2$$

The PBCs in the fill tow direction for the uniaxial tensile load in the *x*-direction can be changed to:

(6a) $$u_{3}^{P}+{\hat{\varepsilon }}_{11}\lambda_{w}/2=u_{3}^{Q},\;v_{3}^{P}=v_{3}^{Q},\;w_{3}^{P}+{\bar{\varepsilon }}_{33}\lambda_{f}/2=w_{3}^{Q}$$

(6b) $$u_{4}^{P}+{\hat{\varepsilon }}_{11}\lambda_{w}/2=u_{4}^{Q},\;v_{4}^{P}=v_{4}^{Q},\;w_{4}^{P}-{\bar{\varepsilon }}_{33}\lambda_{f}/2=w_{4}^{Q}$$

**Figure 4:**
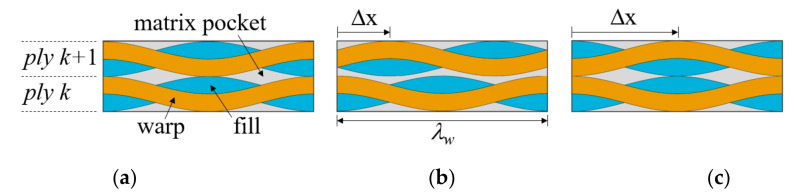
Fiber tow arrangement in the thickness direction: (**a**) in-phase (Δx = 0); (**b**) shifted (Δx = $\lambda_{w}/4$); and (**c**) symmetric (Δx = $\lambda_{w}/2$).

Further reduction of the model size is possible in special cases. When plain weave plies are stacked to have a perfectly in-phase or symmetrical fiber tow arrangement denoted as in-phase and symmetric unit cells, respectively, the tensile tests in the *x*-, *y*-, or *z*-directions can be simulated by using a 1/4-unit cell model, as shown in Figure 3c, exploiting symmetry in the warp and fill tow directions. Figure 4 shows the fiber tow structure in the thickness direction. For these cases, the in-plane direction PBCs under uniaxial tension load in the *x*-direction can be replaced by the following boundary conditions:

*x*-symmetry –

(7a) $$u\left(-\lambda_{w}/4,y,z\right)\;=0,\;u\left(\lambda_{w}/4,y,z\right)\;={\hat{\varepsilon }}_{11}\lambda_{w}/2$$

*z*-symmetry –

(7b) $$w\left(x,y,-\lambda_{f}/4\right)\;=0,\;w\left(x,y,\lambda_{f}/4\right)\;={\bar{\varepsilon }}_{33}\lambda_{f}/2$$

The PBCs in the thickness direction on y = −t/2 and t/2 surfaces are the same as in Equation (4b) for the in-phase fiber tow arrangement. For the symmetric fiber tow arrangement, the PBCs in (4b) can be replaced by the following symmetry condition:

*y*-symmetry –

(7c) $$v\left(x,-t/2,z\right)\;=0,\;v\left(x,t/2,z\right)\;={\bar{\varepsilon }}_{22}t$$

The analysis model size can be further reduced to a 1/16 unit cell shown in Figure 3d for the perfect in-phase or perfect symmetric fiber tow arrangement cases. Here, the *x*- and *z*-symmetry conditions in the positive warp and fill tow surfaces are still exploited, but special PBCs are applied at the *x* = 0 and *z* = 0 surfaces. The boundary conditions at the *x*- and *z*-direction surfaces of the 1/16-unit cell under a uniaxial tensile load in the *x*-direction can be expressed as:

*x*-PBC on *x* = 0 surface –

(8a) $$u\left(0,y,z\right)\;=-u\left(0,-y,z\right),\;v\left(0,y,z\right)\;=-v\left(0,-y,z\right),\;w\left(0,y,z\right)\;=w\left(0,-y,z\right)$$

*x*-symmetry on *x* = $\lambda_{w}$/4 surface –

(8b) $$u\left(\lambda_{w}/4,y,z\right)\;={\hat{\varepsilon }}_{11}\lambda_{w}/4$$

*z*-PBC on *z* = 0 surface –

(8c) $$u\left(x,y,0\right)\;=u\left(x,-y,0\right),\;v\left(x,y,0\right)\;=-v\left(x,-y,0\right),\;w\left(x,y,0\right)\;=w-\left(x,-y,0\right)$$

*z*-symmetry on z = $\lambda_{f}$/4 surface –

(8d) $$w\left(x,y,\lambda_{f}/4\right)\;={\bar{\varepsilon }}_{33}\lambda_{f}/4$$

Note that the PBCs in (8a) and (8c) are elastic analyses only because they do not hold once failure develops. The boundary conditions in the thickness direction are the same as those for the 1/4-unit cell.

In the present study, plain weave unit cells with in-phase, symmetric, and shifted fiber tow arrangements, as shown in Figure 4, were considered. The 1/4-unit cell model was used for cases with in-phase and symmetric fiber tow arrangements and a 1/2-unit cell model for shifted cases. The effect of using different model sizes was studied for the perfect in-phase and symmetric fiber tow arrangement cases. All PBCs were enforced using multi-point constraints.

### 2.3. Finite Element Modeling

In this study, modeling and analyses were performed using the commercial software application ABAQUS (version 6.14) [41]. The fiber tows and matrix pocket of a plain weave composite were discretely modeled using 3D solid elements (C3D8/C3D6). Because fiber tows are orthotropic and the fiber direction constantly varies in the tow path direction, material coordinates are defined for all elements by which material property transformation is performed in the analysis. The material axes are defined as follows: 1—the fiber direction, 2—the transverse direction of the fiber tow cross-section, and 3—the direction perpendicular to both the 1 and 2 directions.

For failure analysis, CZM was used in this study. The considered failure modes were fiber breakage and matrix direction failure in the tow region, cracking in the matrix pocket, and interface separation between the warp and fill tow regions and between the tow and matrix pockets. To account for this, cohesive elements (COH3D8) with corresponding failure properties were inserted between all element interfaces.

The finite element mesh size was determined by estimating the cohesive zone size and then placing a sufficient number of elements within it. The cohesive zone size could be estimated using $l_{cz}=MEG_{C}/T_{max}^{2}$ where E, $G_{C}$, and $T_{max}^{}$ are the elastic modulus, fracture energy, and maximum cohesive traction, respectively, and M is a constant with a value between 0 and 1 [42]. Using the elastic and cohesive properties in Table 2 and Table 3, the cohesive zone size was calculated for each constituent material and failure mode. The minimum $l_{cz}$ was estimated to be 0.106 mm for mode-I failure in the pure matrix pocket. From this, the final mesh density was determined such that approximately 3–4 elements were placed in the estimated minimum $l_{cz}$. In the preliminary analysis, this mesh density was found to produce both converged elastic and failure results.

**Figure 5:**
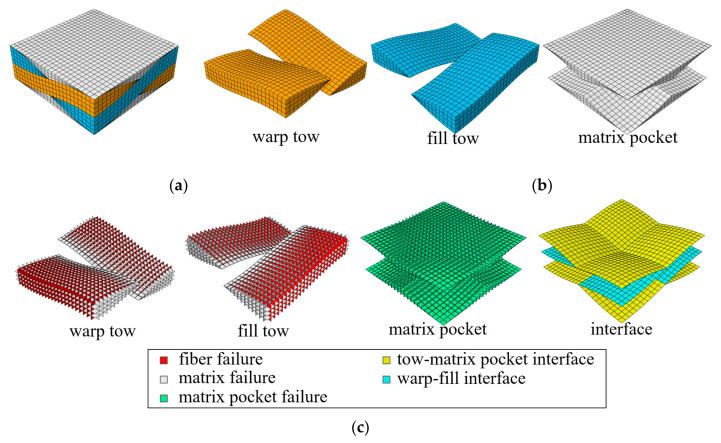
Finite element mesh of single-ply repeating 1/4-unit cell: (**a**) 1/4-unit cell mesh (bulk and cohesive elements); (**b**) bulk elements; and (**c**) cohesive elements.

**Figure 6:**
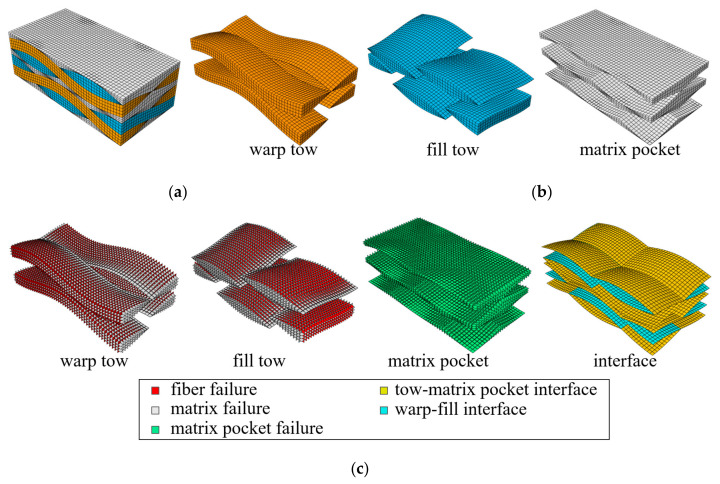
Finite element mesh of two-ply repeating 1/2-unit cell: (**a**) 1/2-unit cell mesh (bulk and cohesive elements); (**b**) bulk elements; and (**c**) cohesive elements.

Figure 5 and Figure 6 show the finite element meshes for a 1/4-unit cell of a single ply ($\lambda_{w}/2\times \lambda_{f}/2\times t_{ply}$) and a 1/2-unit cell of two plies with shift Δ*x* = $\lambda_{w}/4$ ($\lambda_{w}\times \lambda_{f}/2\times 2t_{ply}$), respectively. The latter mesh size was four times larger than that of the former. The break-up views of the bulk elements of the fiber tows and matrix pockets, and cohesive elements with corresponding failure modes are shown. The 1/4-unit cell mesh consisted of 8064 solid elements (C3D8/C3D6) and 22,752 cohesive elements (COH3D8/COH3D6), the total number of nodes being 63,744. The two-ply 1/2-unit cell mesh consisted of 32,256 solid elements and 92,928 cohesive elements, the total number of nodes being 254,976.

### 2.4. Selective Activation of Cohesive Elements

The insertion of cohesive elements between bulk elements increases the computational model size in intrinsic CZM. This is particularly significant when fracture locations and paths are unknown, and thus cohesive elements must be inserted in large regions. The CZM size is still in the manageable range for a single-ply repeating 1/4-unit cell; however, for larger models, such as the two-ply repeating 1/2-unit cell, there are too many degrees of freedom (DOFs), making it impractical to perform conventional CZM analysis, which requires an enormous amount of computational time and memory. Moreover, the insertion of cohesive elements causes an added compliance problem that can affect the accuracy of the final solution. To avoid these problems, extrinsic CZM can be used with explicit solvers in which cohesive elements are inserted adaptively [43,44]. Using extrinsic methods, the above-mentioned problems can be minimized. However, complex data management is required, and fracture criteria are needed to determine where and when to insert cohesive elements.

A modified intrinsic CZM technique called MCZM (MPC controlled CZM) developed in [35,36] can reduce the problem of conventional intrinsic CZM (CCZM) with large insertions of cohesive elements. Using this technique, CZM meshes are initially generated with cohesive elements inserted between all bulk element interfaces. However, prior to analysis, all duplicated nodes inserted are tied by MPC, making all cohesive elements inactive. During analysis, the MPC ties are selectively released as needed to activate the cohesive elements, and the fracture process begins exactly the same as the conventional CZM.

**Figure 7:**
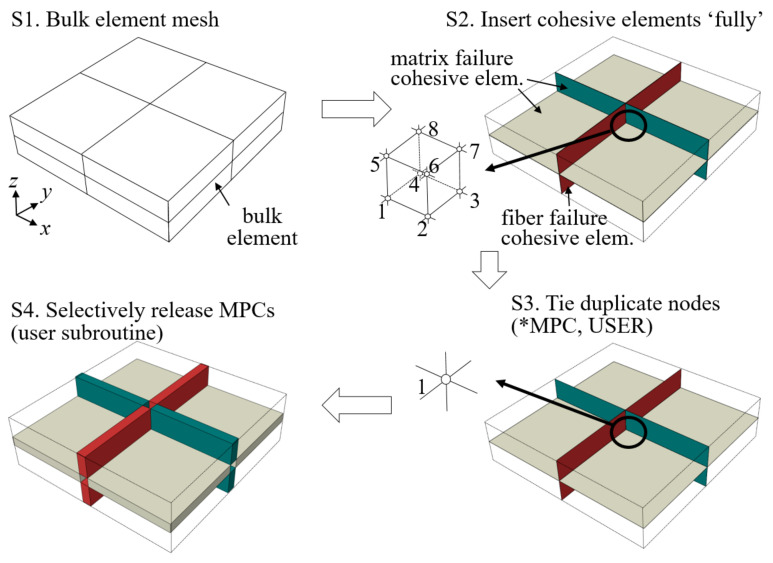
Schematic of MCZM process.

Figure 7 illustrates MCZM process for fiber tows. The process is as follows:

(Step 1) Generate bulk element mesh.

(Step 2) Insert cohesive element for fiber failure, matrix direction failure, and interface separation modes between bulk element interfaces. (Fiber and matrix direction failures are replaced by matrix pocket cracking in pure matrix pocket region.)

(Step 3) Deactivate all duplicate nodes by tying nodes (*MPC, USER).

(Step 4) Start analysis and selectively release MPC ties (user subroutine).

In Step 2 of Figure 7, eight duplicated nodes at the center point are plotted as an example. The distance between duplicate nodes is zero because zero-thickness cohesive elements are used. In Step 3, the duplicate nodes are tied, leaving only one active node. Steps 1–3 are performed prior to the start of the analysis. After Step 3, the total number of active DOFs becomes the same as that of the bulk element-only mesh because all duplicated cohesive nodes are tied by MPC. During Step 4, when the stress states of the bulk elements meet certain conditions, the MPC ties of the connected nodes are released, and the corresponding cohesive elements are activated. In Step 4 of Figure 7, cohesive elements are illustrated as activated with finite deformation.

As the analysis progresses, more MPC ties are released and the total number of DOFs gradually increases but is substantially smaller than that of CCZM because the number of cracks developed and propagated is limited for most cases, significantly reducing the computational resource requirements. For the release condition, the Hashin criterion [45] is used for fiber tow failures, and the von Mises stress condition is used for matrix pocket cracking. Note that the choice of release condition is not important as the actual failure process is governed intrinsically by the traction-separation relationship of CZM. When all MPCs are released at the beginning—that is, when all MPCs are released at zero stresses—MCZM simply becomes CCZM. The MPC tie is implemented using *MPC, USER and the release of MPC ties is performed by a user subroutine in ABAQUS.

## 3. Results and Discussion

### 3.1. Validation of FE Modeling and MCZM

The validation for applying CCZM to predict the failure behavior of plain weave composites was provided in [13], and the validation and efficiency of MCZM were extensively studied in [35,36]. Here, a comparison of results between CCZM and MCZM in the failure analysis of a plain weave composite unit cell is provided to further verify the applicability of MCZM.

**Figure 8:**
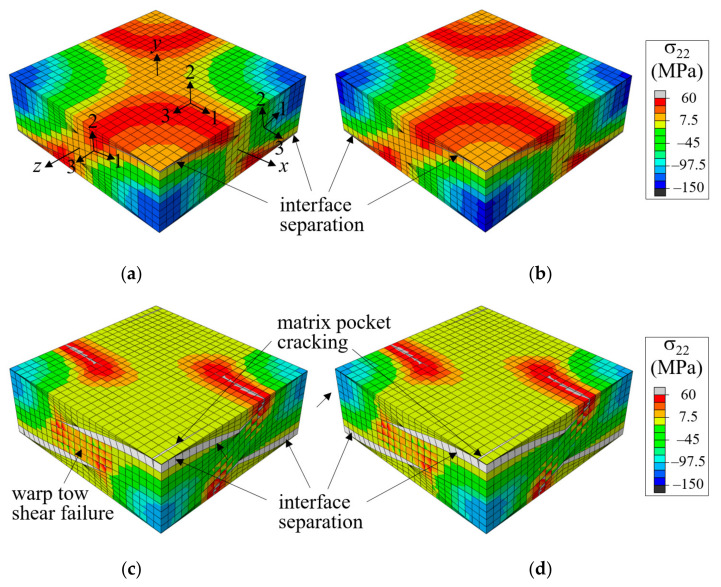
Comparison of $\sigma_{22}$-stress and failure shapes of symmetric plain weave unit cell (*x* = *λ_w_*/2) under tension in the *x*-direction: (**a**) CCZM at ${\hat{\varepsilon }}_{xx}$ = 0.0101; (**b**) MCZM at ${\hat{\varepsilon }}_{xx}$ = 0.0101; (**c**) CCZM at ${\hat{\varepsilon }}_{xx}$ = 0.0102; and (**d**) MCZM at ${\hat{\varepsilon }}_{xx}$ = 0.0102 (deformation scale factor = 2; *xyz*: global coordinates; 123: material coordinates).

**Figure 9:**
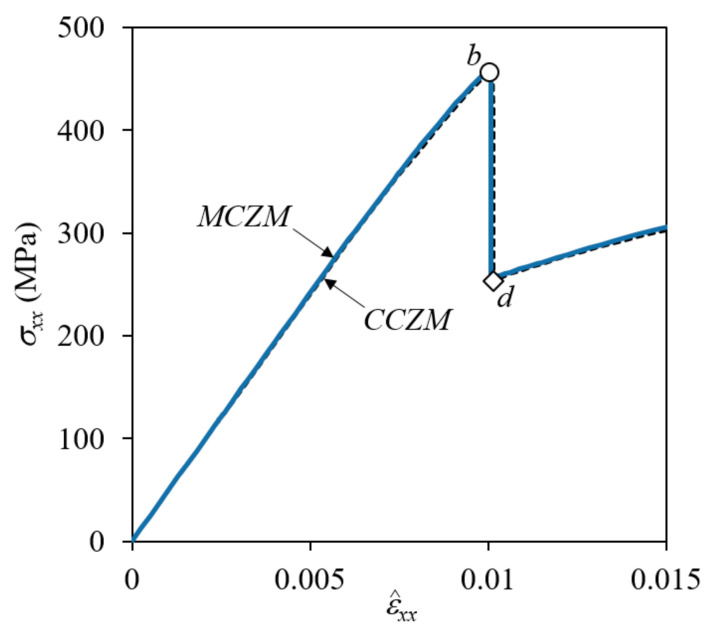
Comparison of nominal stress-strain curves for symmetric plain weave unit cell (*x* = *λ_w_*/2).

Figure 8 and Figure 9 show a comparison of the stress and failure development history and load-displacement curves for the 1/4-plain weave unit cell under uniaxial tensile load. Here, the analysis was performed by specifying the *x*-displacement $\hat{u}={\hat{\varepsilon }}_{xx}\lambda_{w}/2$ for nodes at the *x* = $\lambda_{w}/4$ plane, and the nominal stress was calculated by dividing the *x*-direction reaction force at the *x* = $\lambda_{w}/4$ plane by its surface area. In this case, it was assumed that the plies were stacked to have a symmetric fiber tow arrangement in the thickness direction (i.e., Δ*x* = $\lambda_{w}/2$). In the figure, the material axes are plotted for one element each of the warp tow, fill tow, and matrix pocket, with ‘1’ indicating the fiber direction, as an example. The stress contours and failure development shapes are plotted in Figure 8 for two different applied nominal strain levels of ${\hat{\varepsilon }}_{xx}$ = 0.0101 and 0.0102 (right before and after a large drop in the stress-strain curves marked as *b* and *d*, respectively, in Figure 9). The results obtained by CCZM and MCZM are well matched with negligible differences. Moreover, the nominal stress-strain curves shown in Figure 9 exhibit little difference. These results confirmed that MCZM produced the same results as CCZM in the failure analysis of plain weave composites.

### 3.2. Failure Pattern Dependence on Fiber Tow Arrangement

Failure development patterns differ depending on the fiber tow structure in the thickness direction. This is because the straightening of warp tows under a tensile load in the *x*-direction, the upward, and downward undulating deformation, occurs differently.

Under an *x*-tensile load, the warp tows straighten themselves, producing upward and downward forces that push out the fill tows, trying to generate undulating deformation in the thickness direction. When plies are stacked to have a symmetric fiber tow structure in the thickness direction, the undulating deformation is canceled by the same magnitude and opposite direction deformation of adjacent plies. This causes high tensile σ_22_-stress at the warp tow crown region in which the warp tows meet with warp tows of the adjacent plies and high compressive σ_22_-stress where the fill tows meet.

The first failure occurred at the tip region of the fill tow in the form of matrix direction tension failure when the applied nominal strain was ${\hat{\varepsilon }}_{xx}$ = 0.0597. This was because the warp tow straightening pushed out and forced the fill to deform, thus failing in tensile mode. The matrix failure in the fill tows grew slowly because the fill tows were supported by the surrounding warp tows and matrix pockets. As the tensile load increased to ${\hat{\varepsilon }}_{xx}$ = 0.0076, the failure mode expanded to the entire fill tow region, and the tangential modulus (the slope of the curve) was reduced by approximately 10%. However, this failure in fill tows was still under processed because the fill tows were connected to the warp tow (which transfer most of the applied tensile load) as well as to the matrix pocket material.

As the tensile load increased further, a fiber tow-matrix pocket interface separation failure occurred. The high tensile σ_22_-stress at the warp tow crown region provided the driving force for the mode-I interface separation failure development, as shown in Figure 8b. The interface separation propagated slowly at first and then abruptly when ${\hat{\varepsilon }}_{xx}$ increased from 0.101 (marked *b*) to 0.102 (marked *d*). Figure 8d shows the propagated interface separation as well as the shear failure at the mid-warp tow region and matrix cracking. This sudden propagation resulted in a significant drop in the nominal stress-strain curve, indicating that interface separation was the major failure event for the symmetric fiber tow arrangement case. As the applied nominal strain increased further, the interface separation and warp tow shear failure grew slowly, and the stress-strain curve increased gradually.

**Figure 10:**
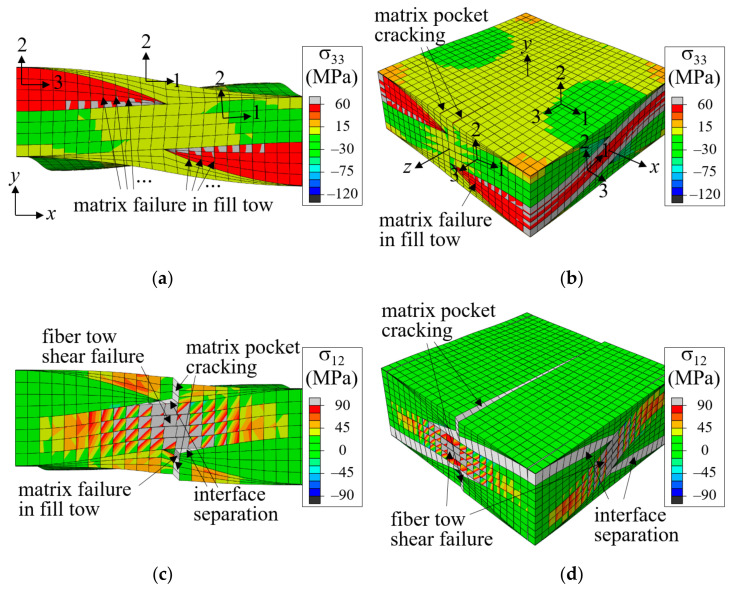
Fracture development process for in-phase stacked plain weave unit cell: (**a**)$\;\sigma_{33}$ at ${\hat{\varepsilon }}_{xx}$ = 0.00794; (**b**)$\;\sigma_{33}$ at ${\hat{\varepsilon }}_{xx}$ = 0.0121; (**c**)$\;\sigma_{12}$ at ${\hat{\varepsilon }}_{xx}$ = 0.0180; and (**d**)$\;\sigma_{12}$ at ${\hat{\varepsilon }}_{xx}$ = 0.0189 (Deformation scale factor is 10 for (**a**) and 2 for (**b**–**d**)).

**Figure 11:**
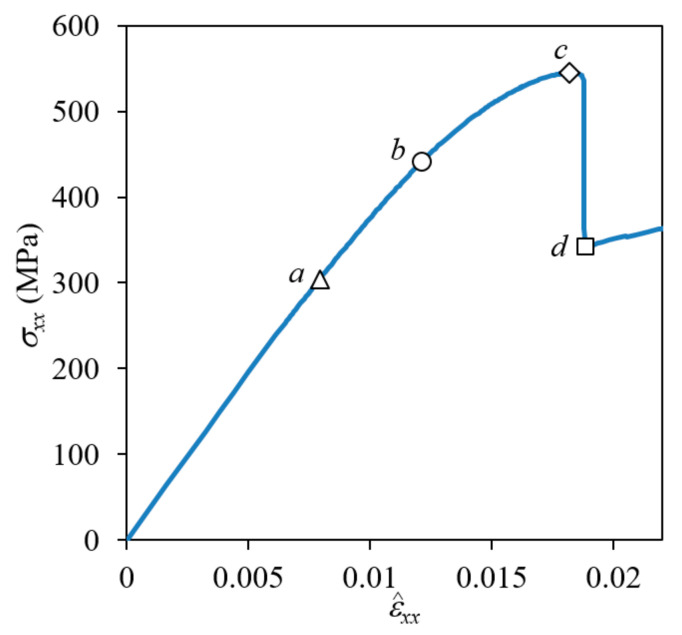
Nominal stress-strain curve for in-phase plain weave unit cell. (Markers *a*–*d* match to the fracture states in Figure 10.)

Figure 10 shows the fracture development process of a plain weave unit cell when the fiber tow structure in the thickness direction is in-phase (Δx = 0), with (a) and (c) in the positive *z*-boundary surface view and (b) and (d) in a bird’s-eye view. The distribution of σ_33_ is plotted in (a) and (b), and σ_12_ is plotted in (c) and (d). The nominal stress-strain curve is plotted in Figure 11, where symbols *a*-*d* correspond to the points in Figure 10a–d.

In the in-phase case, fiber tows are arranged to have warp and fill tows alternately in the thickness direction, and the upward and downward deformation of warp tows occurred in the same direction and did not interfere with each other. As a result, a large amount of undulating deformation occurred because of the straightening-shear coupling of warp tows under the *x*-tension load, as can be seen in Figure 10a. Thus, the in-phase case is characterized as shear-dominant, and its failure behavior is different from that of the symmetric case. The matrix failure in the fill tow started when ${\hat{\varepsilon }}_{xx}$ = 0.00439. Subsequently, as the tensile load increased, the entire fill tow region entered the failure process, as shown in Figure 10a at ${\hat{\varepsilon }}_{xx}$ = 0.00794 and Figure 10b at ${\hat{\varepsilon }}_{xx}$ = 0.0121 (marked as *a* and *b*, respectively, in Figure 11). At this stage, the matrix pocket was also under high σ_11_ tensile and σ_12_ shear stresses (which is not seen because of the differences in local coordinates). The matrix failure in the fill tows appeared to be more severe near the region where the crimp angle of the warp tows was largest, and thus the upward or downward push-stretched deformation due to the straightening of the wavy warp tow was concentrated. While matrix failure developed extensively in the entire fill tow region, this failure process was not fully completed for most parts because the fill tows were attached to the surrounding warp tows and matrix pockets. At ${\hat{\varepsilon }}_{xx}$ = 0.0121, cracks began developing in the matrix pocket region as shown in Figure 10b (marked as *b* in Figure 11) caused by high tensile and shear stresses. Initially two matrix pocket cracks occurred, competing with each other. However, as the load increased, only one propagated while the other did not. As the applied nominal strain increased further, the warp tow started to fail in shear, as shown in Figure 10c at ${\hat{\varepsilon }}_{xx}$ = 0.0180 (marked as *c* in Figure 11). The shear failure process in the warp tow was slow because the shear fracture energy was relatively large. Finally, the interface separation occurred and propagated extensively within a short increase in the applied nominal strain as shown in Figure 10d.

When plies were repeatedly stacked in two plies with a phase shift between plies of Δ*x* = λ*_w_*/4, a more complicated failure pattern was observed, which is a mixed version of both of two extreme cases: the symmetric and in-phase arrangements.

**Figure 12:**
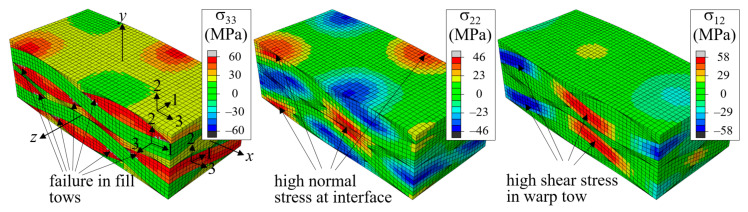
Stress distribution for two-ply shifted unit cell under *x*-tension at ${\hat{\varepsilon }}_{xx}$ = 0.006 (deformation scale factor = 5).

Figure 12 shows the stress distributions for the two-ply shifted unit cell when the applied strain was ${\hat{\varepsilon }}_{xx}$ = 0.006. The entire fill tow region has high tensile σ_33_ stress distribution. The first failure occurred at ${\hat{\varepsilon }}_{xx}$ = 0.00489 at the fill tow edge region as indicated in the left figure. As the tensile load increased, the entire fill tow region entered in failure process, however, this failure was contained because the fill tows were supported by warp tows and matrix pocket material. High tensile σ_22_ stress occurred at the region where the warp tows of adjacent plies are in a close distance and high σ_12_ stress occurred in the warp tows as indicated in the center and right figures, respectively. Thus, failures were expected to occur in these regions when the tensile load increased further.

**Figure 13:**
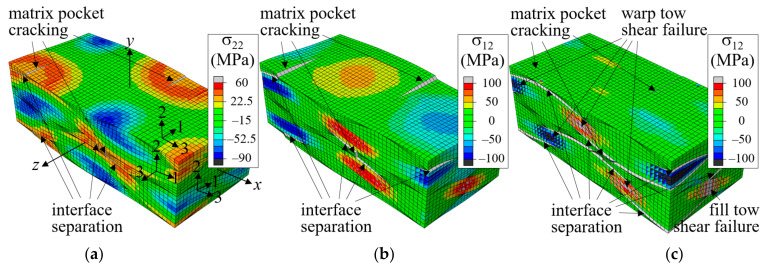
Fracture development process for two-ply shifted unit cell: (**a**) $\sigma_{22}$ at ${\hat{\varepsilon }}_{xx}$ = 0.00969; (**b**) $\sigma_{12}$ at ${\hat{\varepsilon }}_{xx}$ = 0.0136; and (**c**) $\sigma_{12}$ at ${\hat{\varepsilon }}_{xx}$ = 0.0138 (Deformation scale factors are 5, 3, and 1 for (**a**–**c**), respectively).

**Figure 14:**
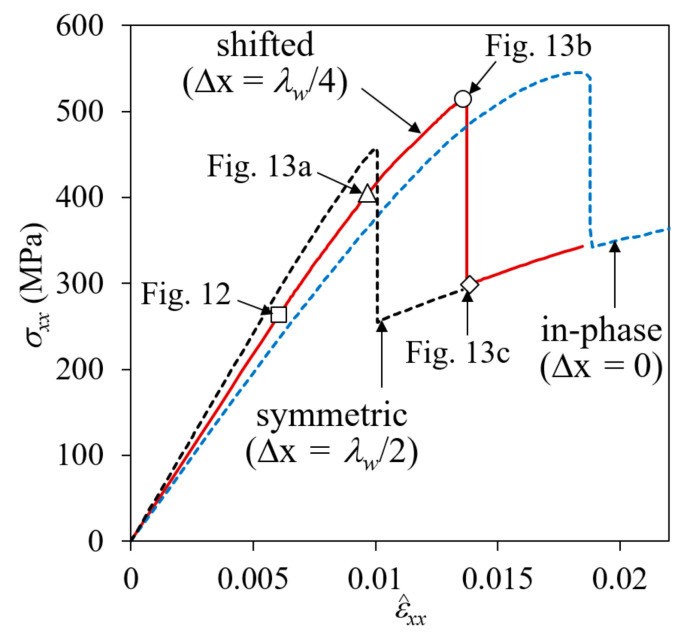
Nominal stress-strain curve for two-ply shifted unit cell.

Figure 13 shows snap shots of the failure development history. The nominal stress-strain curve is shown in Figure 14, in which the failure development points plotted in Figure 12 and Figure 13 are indicated. The curves for the symmetric and in-phase cases are also plotted for comparison. As mentioned previously, the first failure occurred in fill tows in matrix tensile mode at ${\hat{\varepsilon }}_{xx}$ = 0.00489, followed by matrix pocket cracking and interface separation at ${\hat{\varepsilon }}_{xx}$ = 0.00671 in the region where the distance between the warp tows of adjacent plies was the smallest. The interface separation initiated in tensile dominated mode, but the failure mode switched to normal-shear mixed mode and grew slowly, as shown in Figure 13a,b. This slow and gradual growth resulted in a large nonlinearity in this range of the stress-strain curve.

Subsequently, the failure growth reached a critical point at approximately ${\hat{\varepsilon }}_{xx}$ = 0.0136 and an extensive interface separation failure growth occurred, as shown in Figure 13c, resulting in a sharp drop in the stress-strain curve. The separation failure occurred mostly at the warp tow-matrix pocket interfaces, and not at the warp-fill and fill-matrix pocket interfaces, as the shear stress was relatively small and the normal stress perpendicular to the interface (σ_22_) was compressive. The extensive interface separation caused the fiber tows to deform freely, causing shear failure to start at the warp and fill tows.

In Figure 14, the nominal stress-strain curve of the symmetric case exhibited the most stiffness initially, showing almost linear behavior until a large drop was observed. In contrast, the curve of the in-phase case exhibited the least stiffness initially, before becoming non-linear, owing to the development of gradual shear failure in the warp tows. Compared to the symmetric case, the initial stiffness of the in-phase case was 18.2% less, but the maximum nominal stress was 21.5% more. The nominal stress-strain curve of the shifted case (Δ*x* = λ*_w_*/4) fell between the two, showing an 8.2% decrease and 13.9% increase in initial stiffness and maximum stress, respectively, compared to the symmetric case.

The difference in the failure pattern between cases with different fiber tow arrangements is due to the interaction of adjacent plies. As a tensile load is applied in the *x*-direction, the warp tows straighten. When the fiber tow structure is in-phase, this straightening deformation occurs repeatedly in adjacent plies. This deformation occurs relatively freely, producing large shear deformation that results in shear-driven failure modes. By contrast, when the fiber tow structure is symmetric, the straightening deformation is resisted by the vertically opposite direction deformation of the adjacent plies. This causes high out-of-plane tensile stresses in the region where the adjacent warp tows meet, resulting in IS. For the shifted case with Δ*x* = *λ_w_*/4, the deformation is between the other two cases, demonstrating a mixed failure pattern.

### 3.3. Effect of Defect for Symmetric and in-Phase Plain Weave Unit Cells

**Figure 15:**
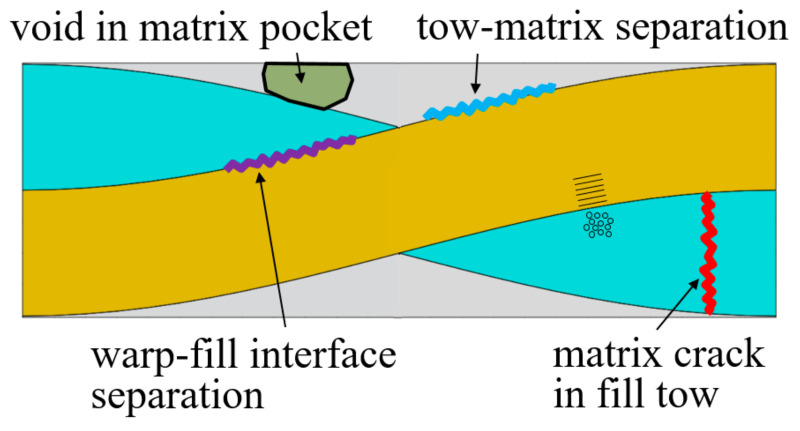
Types of defects in plain weave textile composites.

Textile composites have diverse manufacturing defects as reported in [15,16,17,18,19]. These defects can be summarized as voids in the matrix pockets, cracks in the warp and fill tows, and interface separation as illustrated in Figure 15.

**Figure 16:**
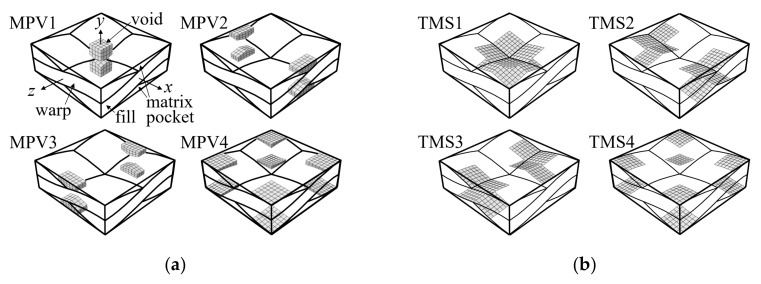

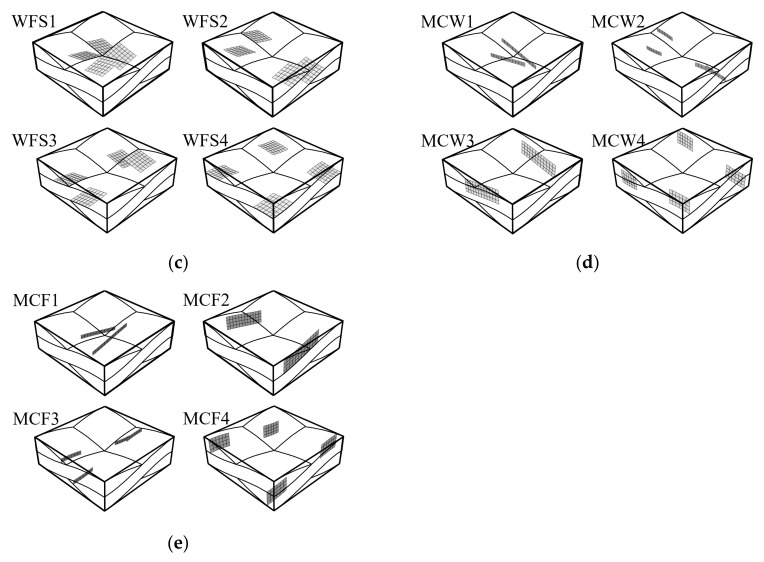
Definition of defect codes by types and location: (**a**) matrix pocket void; (**b**) tow and matrix pocket separation; (**c**) warp tow and fill tow separation; (**d**) matrix direction crack in warp tow; and (**e**) matrix direction crack in fill tow.

Figure 16 shows the types and locations of defects considered for the in-phase and symmetric cases. While the sizes and distribution of defects are random in nature, different types of defects occurring simultaneously, predetermined defect shapes and locations are assumed herein. The types of defects and locations are classified using defect codes consisting of key words and region numbers, respectively, as shown in Figure 16. The considered defects are: voids in matrix pocket (MPV), tow-matrix pocket separation (TMS), warp-fill separation (WFS), and matrix cracks in the warp (MCW) and fill tows (MCF). Region 1 is defined as the region where all edges of the warp and fill tows cross, and Region 4 is defined as the crown parts of the warp and fill tow wave. Region 2 is defined as the region where the warp tow edge crosses the fill tow, and vice versa for Region 3.

In the numerical model, the defects were modeled by the deleting bulk and cohesive elements in the defect region. At the surfaces with deleted cohesive elements, contacts were defined to avoid penetration of bulk elements. Each defect type was assumed to occur separately. Eight separation defects were placed between the fiber tow-matrix pocket interfaces for the TMSs, and four separation defects were placed between the warp-fill tow interfaces for the WFSs. Each separation defect area was 0.199 × 0.199 mm^2^. The length of the MCWs and MCFs was 0.199 mm, but the heights were 0.033 mm for MCW1, MCW2, MCF1, and MCF3 and 0.12 mm for MCW3, MCW4, MCF2, and MCF4. The MCWs and MCFs were placed at a distance of 0.0645 mm from the center and edge surfaces. The length, width, and thickness of the MPV defects were varied to have similar void volumes. The void fractions for the MPV1, MPV2, MPV3, and MPV4 models were 2.42, 2.64, 2.64, and 2.75%, respectively.

**Figure 17:**
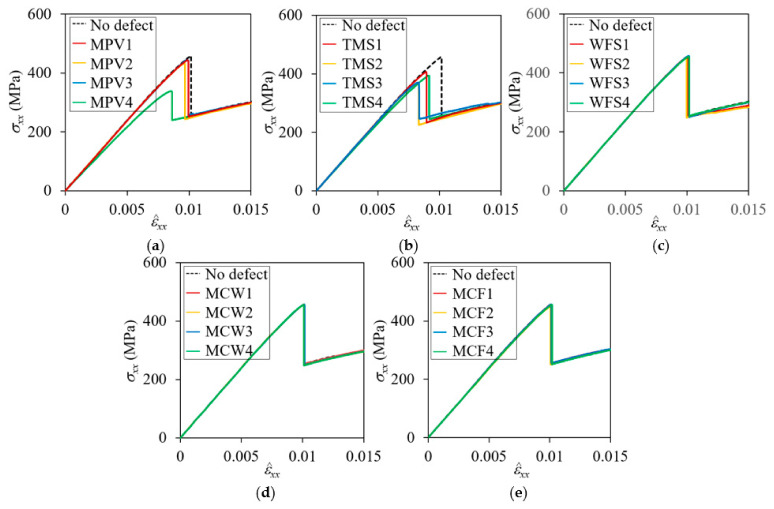
Effect of defects on nominal stress-strain curves for symmetric unit cell: (**a**) matrix pocket void; (**b**) tow and matrix pocket separation; (**c**) warp tow and fill tow separation; (**d**) matrix direction crack in warp tow; and (**e**) matrix direction crack in fill tow.

**Figure 18:**
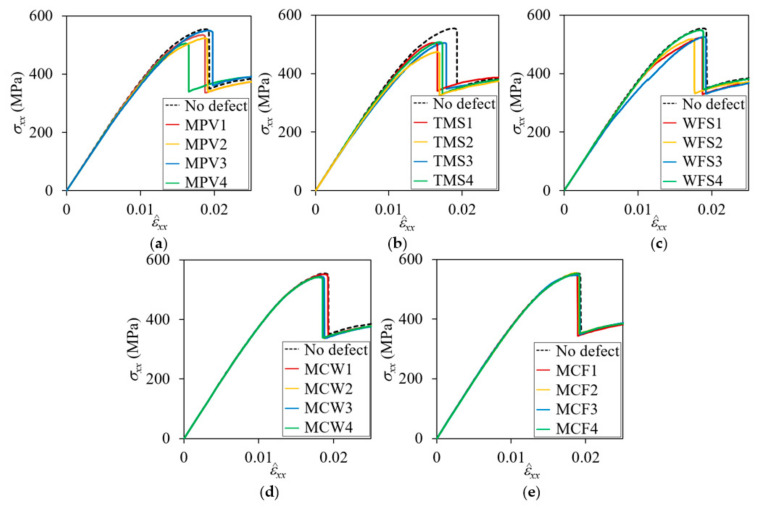
Effect of defects on nominal stress-strain curves for in-phase unit cell: (**a**) matrix pocket void; (**b**) tow and matrix pocket separation; (**c**) warp tow and fill tow separation; (**d**) matrix direction crack in warp tow; and (**e**) matrix direction crack in fill tow.

Figure 17 and Figure 18 show the nominal stress-strain curves of the plain weave unit cell models with defects for the symmetric and in-phase cases, respectively. Table 4 summarizes the reduction of peak nominal stresses compared to the pristine peak stresses. The effect of defects appears differently depending on the defect types, location, and fiber tow arrangement. It was found that MPV and TMS defects had large effects on the symmetric cases and MPV, TMS, and WFS defects on the in-phase cases.

**Figure 19:**
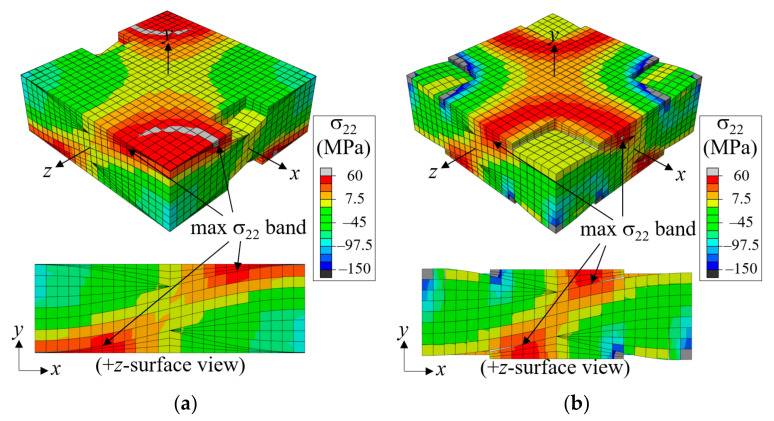
Comparison of σ22 distribution for symmetric unit cells at ${\hat{\varepsilon }}_{xx}$= 0.008; (**a**) MPV2; and (**b**) MPV4. (Deformation scale factor = 2).

For the symmetric case, the MPV4 defect (MPV defect located in Region 4) was found to have the most significant effect. Region 4 is where the crown part of the warp tow meets with that of the next layerin the thickness direction (as with the fill tows), and the removal of the matrix in this region made the straightening of the warp tows occur relatively more easily than in other MPV cases. Moreover, the fill tow surface comes into contact with that of adjacent layers during deformation, and thus the matrix direction failure in the fill tows develops early, as shown in Figure 19b showing the σ_22_-distributions of MPV4 at ${\hat{\varepsilon }}_{xx}$ = 0.008. As a result, a much smaller peak stress occurred versus the other MPV cases. Compared to the pristine case, the reduction in the peak stress was 25.74%, which is particularly noticeable considering that the void fraction was 2.75%. By contrast, MPV1-3 exhibited relatively small reductions in peak stresses. In Figure 19, the progression of the maximum σ_22_-bands along the fiber tow-matrix pocket interface (which coincides with the front line of the fracture process zone) of MPV2 was delayed compared to that of MPV4. The delayed progression of the fracture process zone of MPV2 resulted in a delayed peak stress load and thus a smaller reduction in peak stress compared to MPV4.

**Figure 20:**
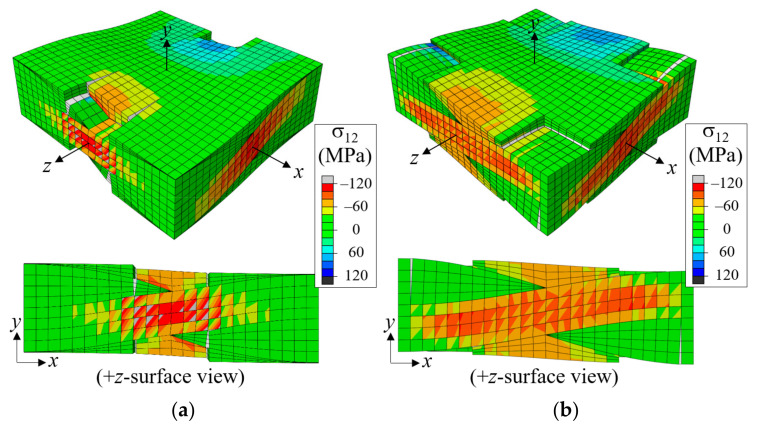
Comparison of σ12 distribution for symmetric unit cells at ${\hat{\varepsilon }}_{xx}$ = 0.0129; (**a**) MPV3; and (**b**) MPV4. (Deformation scale factors are 5 for iso-view and 2 for front z-surface view).

For the in-phase case, the effect of the void was largest for MPV4 with the nominal strain at the peak stress being 14.2% smaller than the pristine case. Owing to the highly nonlinear stress-strain curve, the reduction in the peak stress was just 7.35%. Other MPV cases showed reduced effects. In particular, MPV3 showed almost no reduction in peak stress and a larger strain at peak stress. This was because of the shear stress relief at the fiber tow-matrix pocket interface in the other regions. That is, the failure of the in-phase plain weave unit cell was σ_12_-shear stress dominated, and the warp tow in Region 3 had the highest σ_12_-shear stress distribution under tensile load in the *x*-direction. Consequently, voids in Region 3 helped localize the shear deformation of the warp tow in that region, effectively releasing shear stress at the fiber tow-matrix pocket interface in the other regions, as shown in Figure 20.

TMS defects had a slightly different effect pattern. In the symmetric case, all TMS defects had a significant impact. In particular, TMS2 and TMS3 defects showed significant effects, reducing peak stresses by 18.95% and 18.81%, respectively. This was contrary to the perception that TMS4 would have the largest effect because the separation starting region was Region 4 for the pristine case. The reason that TMS2 and TMS3 had larger effects than TMS4 was due to the mode change of the interface failure. For the symmetric case, the interface failure occurred predominantly in the σ_22_ opening mode in Region 4. However, in the other regions, the inclination angle of the fiber tow-matrix pocket interface increased, which resulted in the stress distributions with a decreased σ_22_ and an increased σ_12_; thus, the interface failure occurred in the opening-shear mixed-mode. Because the fracture energy of the shear mode was larger than that of the opening mode, the interface failure in Regions 2 and 3 required more energy to be provided than that in Region 4. As a result, the interface separation could occur much more easily for TMS2 and TMS3 defects than TMS4. For TMS1, the effect was reduced because the separation at Regions 2 and 3 had to pre-occur.

**Figure 21:**
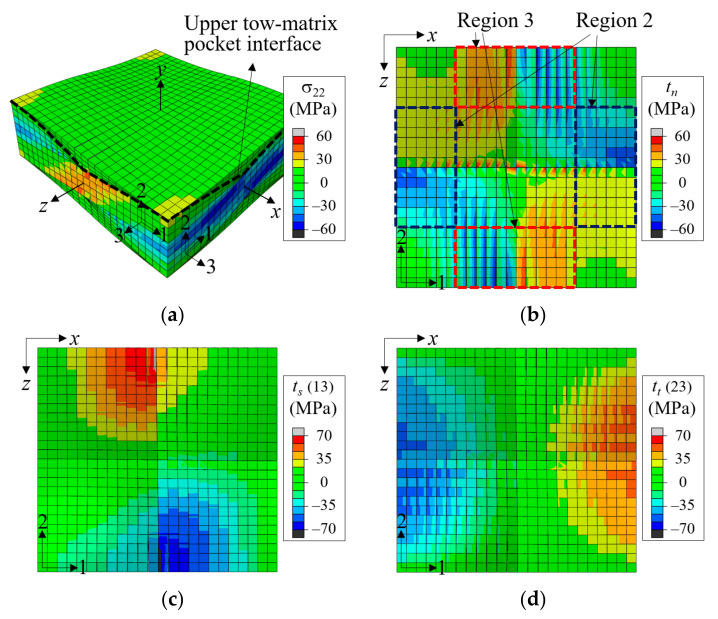
Distribution of σ_22_ of bulk elements and tractions of cohesive elements at the upper tow-matrix pocket interface for in-phase plain weave unit cell without defect at ${\hat{\varepsilon }}_{xx}$ = 0.0073: (**a**) σ_22_; (**b**) normal traction (*t_n_*); (**c**) 13-shear traction (*t_s_*); and (**d**) 23-shear traction (*t_t_*), (deformation scale factor = 5).

In the in-phase cases, the TMS defects also markedly affected the stress-strain curves and peak stresses. The TMS1, TMS3, and TMS4 cases showed reductions of 8.46–8.9% in peak stress. However, in TMS2, the reduction was 14.44%, indicating that Region 2 was the most sensitive location for the tow-matrix separation defect. This can be explained from Figure 21, which shows the distribution of σ_22_ for the bulk elements and normal and shear tractions at the tow-matrix pocket interface for the pristine case without defect at ${\hat{\varepsilon }}_{xx}$ = 0.0073. As shown in Figure 21a, the warp tows straightened under *x*-tensile load while the fill tows became wavier. Because of this, positive and negative σ_22_ stresses developed in the warp tows and fill tows, respectively, where the inclination angle was largest. As a result, the normal traction at the warp tow-matrix pocket boundary was larger in Region 3 than in Region 2, as shown in Figure 21b. These regions also developed large shear stresses (and thus large shear tractions) at the tow-matrix pocket interface, but Region 3 had a larger shear traction distribution than Region 2. Combined, these made the interface separation easier in Region 3 than in Region 2. Consequently, the separation failure developed more easily for TMS2 with the interface separation defect in Region 2 than other cases.

WFS defects did not have any impact on symmetric cases because the warp and fill tows compressed each other, and there were not enough shear stresses developed due to symmetry. However, WFS defects affected the failure behavior for in-phase cases with large shear deformations. WFS1-3, with the warp-fill separation defect placed at the region with a maximum inclination angle either of warp tow (WFS3) or fill tow (WFS2), or both (WFS1), showed a 5.17–6.58% reduction in peak stress. However, the effect was negligible for WFS4 because the warp and fill tows were symmetric in their running directions in Region 4.

**Figure 22:**
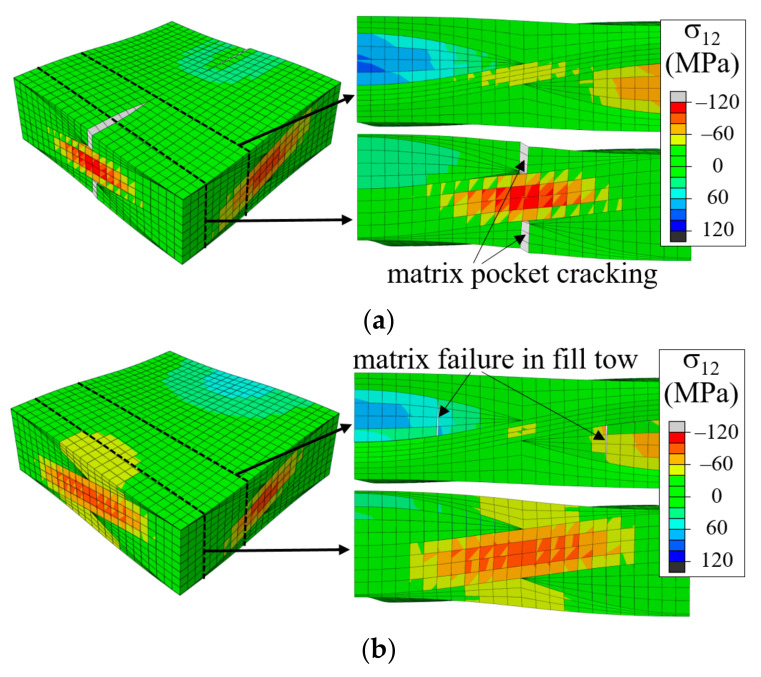
Comparison of failure development of in-phase unit cell with warp-fill separation at ${\hat{\varepsilon }}_{xx}$ = 0.009: (**a**) WFS3; and (**b**) WFS2 (deformation scale factor = 5).

In Figure 18c, the in-phase WFS3 showed a large slope change that occurred early in the process. This was because the matrix pocket failure propagated early from the pre-separated fill tow edge line, as shown in Figure 22a. As the tensile load increased, the matrix crack fully propagated in the *z*-direction. Subsequently, shear failure started in the warp tows, followed by tow-matrix pocket interface separation, resulting in a significant drop in the stress-strain curve. By comparison, the WFS2 case had matrix cracks developed in the fill tows along the edge line of the warp-fill separation defect, as shown in Figure 22b. However, this crack did not propagate further. As the load increased, the tow-matrix pocket separation started at Region 3, not Region 2, because the thicker warp tow in region 3 provided a larger shear load. While showing different failure development sequences, the peak stress reduction of WFS3 was similar to that of WFS2 and WFS1.

MCW and MCF defects were found to be unimportant. For both symmetric and in-phase plain weave unit cells, the stress-strain curves with these defects were almost identical to those without defects, and the reduction in peak stress was negligible.

### 3.4. Effect of Defect for Two-Ply Shifted Plain Weave Unit Cell

Next, the effect of defects was investigated using a two-ply shifted plain weave configuration. Because the matrix pocket void and tow-matrix pocket separation defects were found to be important in the symmetric and in-phase configurations, analyses were performed for these two defect types.

**Figure 23:**
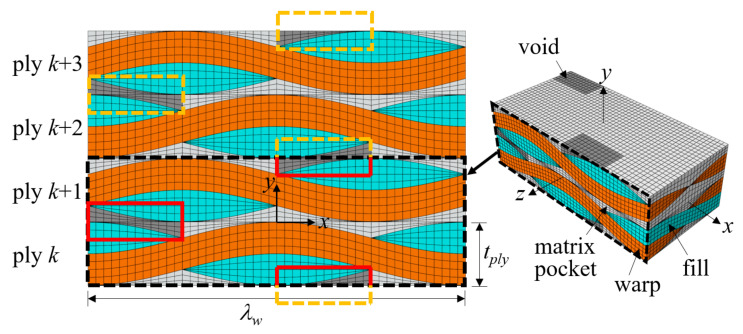
Definition of void type from regional pattern of matrix pocket for two-ply shifted plain weave unit cell (Δx = $\lambda_{w}/4$ ).

The voids were allocated to study the effect of void location with respect to the tow arrangement in the thickness direction. Because the warp tow edge region was found to be less important in terms of the effect of void defects in the symmetric and in-phase cases, voids were placed between the tows along the mid lines of the warp tow paths. The matrix pocket between the fiber tows can be divided into three regions: between warp tows, between fill tows, and between warp and fill tows. In Figure 23, the gray colored matrix pocket region that is located between the fill tows in the thickness direction is shown as an example. In this figure, an additional two-ply was plotted to show the repeating pattern in the thickness direction. These regional patterns repeat following the periodicity vectors ${\vec{r}}_{1}$, 2${\vec{r}}_{2}$, ${\vec{r}}_{3}$, ${\vec{r}}_{4}$, and ${\vec{r}}_{5}$ in (2) and (5). Moreover, the same pattern is obtained by moving the gray region by $\lambda_{w}/2$ in the warp direction and rotating the unit cell by 180° against the *z*-axis.

**Figure 24:**
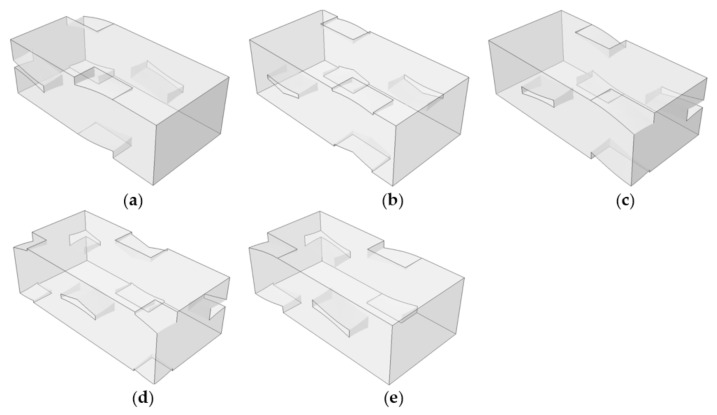
Definition of void defects for two-ply shifted plain weave unit cell: (**a**) MPV21; (**b**) MPV22; (**c**) MPV23; (**d**) MPV24; and (**e**) MPV25.

In this study, five types of void defects were considered based on a repeating pattern of the fiber tow and matrix pocket structures. These were named MPV21-25, as shown in Figure 24. MPV21 corresponds to the void defect shown in Figure 23. The MPVs were named according to how the voids were placed between the warp and fill tows in the thickness direction; MPV21 was placed between 100% fill tows, MPV25 between 100% warp tows, MPV23 between 50% warp tows and 50% fill tows, MPV22 between 75% fill tows and 25% warp tows, and MPV24 between 25% fill tows and 75% warp tows. The above boundary percentage was thought to be important because the interaction of the warp and fill tows and the matrix pocket depends on the arrangement of the fiber tows in the thickness direction. Subsequently, the boundary of these voids in connection to the warp and fill tows was defined as TMS21-25 defects to compare the effects of the MPV and TMS defects. The void fractions of MPV21-25 were all 3%, and the defect areas of TMS21-25 were 0.398 mm × 0.398 mm per interface.

**Figure 25:**
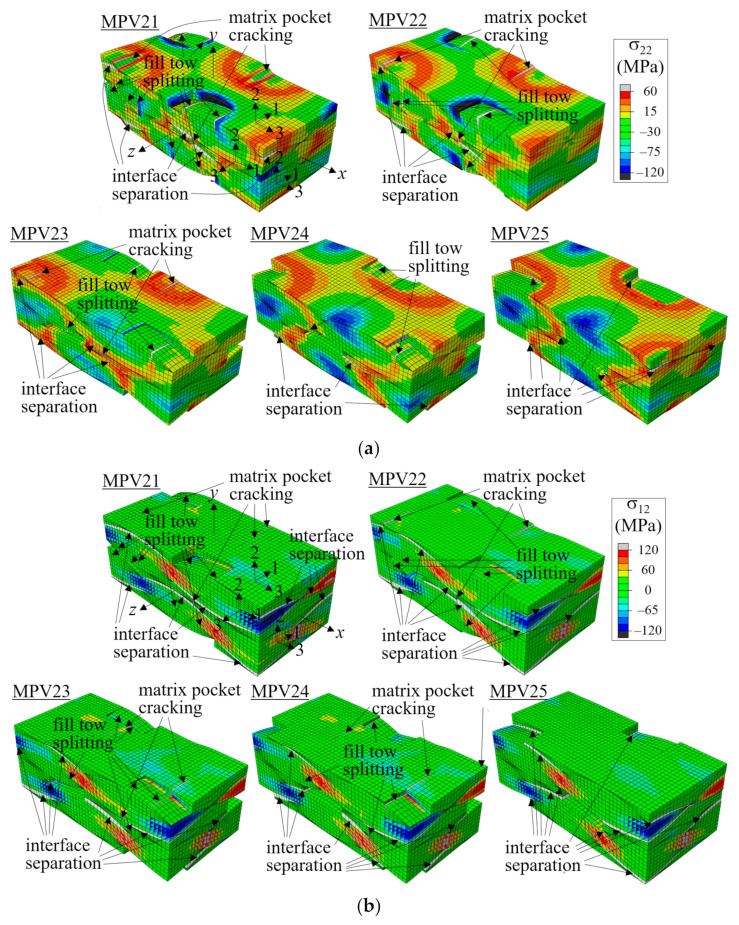
Failure development of two-ply shifted model with MPV defect: (**a**) σ_22_ stress distribution at ${\hat{\varepsilon }}_{xx}$ = 0.00969 (deformation scale factor = 5); and (**b**) σ_12_ stress distribution at ${\hat{\varepsilon }}_{xx}$ = 0.0138 for MPV21,23-25 and ${\hat{\varepsilon }}_{xx}$ = 0.015 for MPV22 (deformation scale factor = 1).

**Figure 26:**
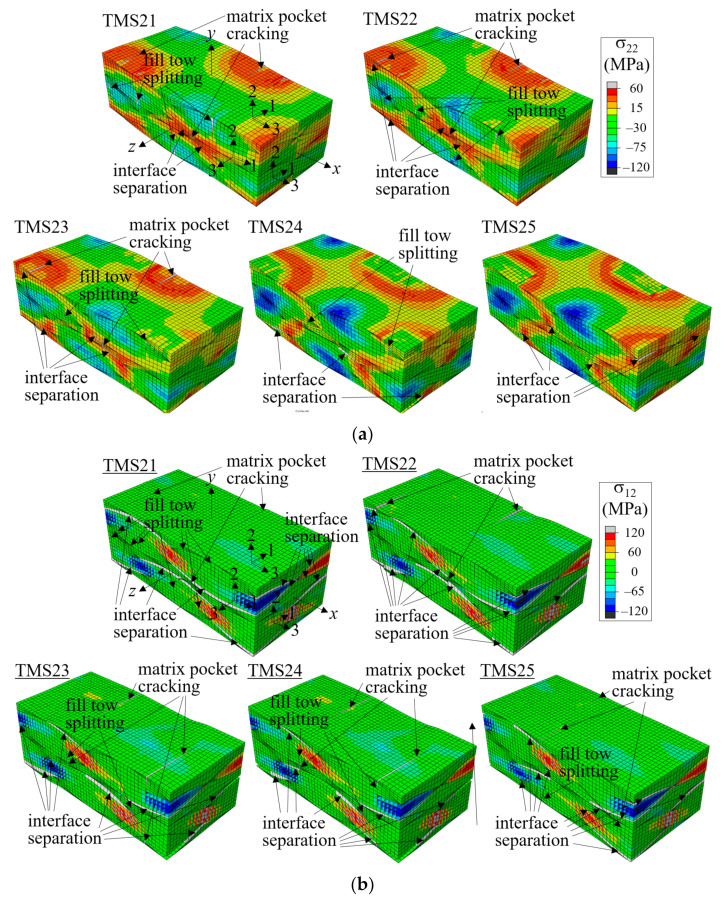
Failure development of two-ply shifted model with TMS defect: (**a**) σ_22_ stress distribution at ${\hat{\varepsilon }}_{xx}$ = 0.00969 (deformation scale factor = 5); and (**b**) σ_12_ stress distribution at ${\hat{\varepsilon }}_{xx}$ = 0.0138 (deformation scale factor = 1).

Figure 25 and Figure 26 show the failure development plots for two-ply shifted unit cells with MPV and TMS defects, respectively, at two different applied nominal strain levels. (See Figure 13 for the failure shapes of the no-defect case.) The material axes for the warp tow, fill tow, and matrix pocket are indicated for one element per material. As did in the no-defect case and in the symmetric and in-phase cases, the initial failure started at the fill tows in the form of matrix direction tensile failure driven by high σ_33_-stress in the fill tows. With MPV and TMS defects, the failure in the fill tows became more severe and fully developed to split the fill tows, as shown in Figure 25a and Figure 26a when the applied nominal strain was ${\hat{\varepsilon }}_{xx}$ = 0.00969. This was because the defects caused more straightening deformation of the warp tows and more push-out deformation of the fill tows. The splitting failure of the fill tows was most prominent for MPV21 and TMS21, whereas it was not found for MPV25 and TMS25. This was because, for MPV21/TMS21, the defects were located between 100% fill tows or on fill tow surfaces and at which compressive σ_22_ was acting in the no-defect case, the free surfaces making the splitting of fill tows by high σ_33_ much easier. By contrast, for MPV25 and TMS25, the defects were solely between the warp tows or warp tow surfaces, and no fill tow splitting was developed.

In other cases, the extent of fill tow splitting decreased as the defect number increased because the percentage of free surfaces formed by the defects decreased and σ_22_ changed from compressive to tensile stress, in that order. The matrix pocket cracking and interface separation failure developed at a similar location, which was the matrix pocket and interface between two adjacent warp tows, similar to the no-defect case shown in Figure 13. However, for MPV24-25 and TMS24-25, these failures were partially removed by the defects. When the location was the same, MPV and TMS defects showed similar failure development patterns, while the extent of failure was larger for MPV than TMS, which was as expected.

Figure 25b and Figure 26b are shown when the applied nominal strain is ${\hat{\varepsilon }}_{xx}$ = 0.0138, except MPV22 for which ${\hat{\varepsilon }}_{xx}$ = 0.015. All cases show the same failure pattern, which is the same as that with no defect as shown in Figure 13c, indicating that while the defects changed the early failure development pattern (and the stress-strain curves and peak stresses), they did not significantly affect the final failure pattern. In the figure, all cases developed extensive interface separation and matrix pocket cracking as well as fill tow splitting failure.

**Figure 27:**
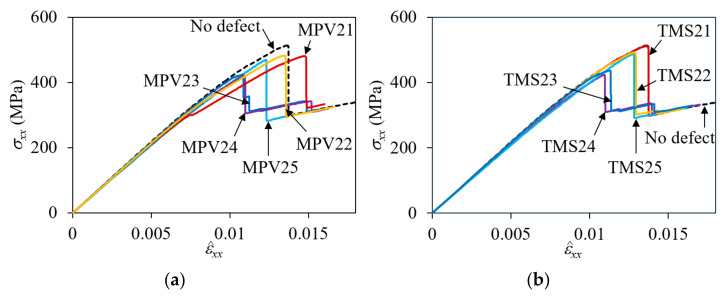
Effect of defects on nominal stress-strain curves for two-ply shifted plain weave unit cell: (**a**) MPV; and (**b**) TMS.

The nominal stress-strain curves for the two-ply shifted unit cell with MPV and TMS defects are shown in Figure 27. Table 5 summarizes the peak nominal stresses and percentage reduction compared to the no-defect configuration. As in the symmetric and in-phase cases, the effect of defects was different depending on the location of the defects. For both types of defects, the maximum impact occurred when the defect was placed not at the location where the interface separation failure was initiated for the no-defect case, but at the location next to it. This was because the propagation of the interface separation slowed when it tried to jump from the concave warp-fill crossing interface to the warp-only convex interface, the defect at that location helping the propagation of the interface separation failure. The reduction in the peak stress was 18.36% for MPV24 and 17.61% for TMS24. MPV23 and TMS23, with defects placed at the next distant locations from the separation initiation point, were also significantly affected for the same reason, with reductions of 17.25% and 15.01%, respectively.

One can see that the stress-strain curve of MPV21 was highly affected and showed an early slope change and increased failure strain. This was because the voids were placed above and below the initial interface separation location, which freed up the up-and-down deformation of the warp tows, accelerating the timing of the initial separation. The large warp tow deformation also caused fill tow splitting and matrix pocket cracking, which together with the early interface separation failure reduced the stiffness. However, the early initiation of interface separation did not facilitate the following propagation. The failure strain at which extensive interface separation occurred increased by 8.2% with a 6.24% reduction in peak stress compared to the no-defect case. By contrast, the effect was negligible for TMS21 because the matrix pocket between the interface resisted the up-and-down deformation of the warp tows.

## 4. Conclusions

In this paper, the progressive failure behavior of plain weave textile composites with defects was studied by 3D finite element unit cell analysis and CZM, fully accounting for all failure modes. The following conclusions were drawn from the unit cell analyses of the plain weave composites under tensile load in the *x*-direction.

It was found that, for the plain weave configuration considered in this study, the first failure occurred in fill tows in tensile mode perpendicular to the loading direction for all cases. However, this failure mode was contained because the fill tows were supported by warp tows and matrix pockets. The main failure mode was fiber tow-matrix pocket interface separation, which resulted in a sharp drop in the nominal stress-strain curves.

It was also found that the progressive failure behavior was dependent on the fiber tow arrangement in the thickness direction. For the symmetric unit cell, the tensile interface failure caused by the straightening of warp tows was the main failure mode, and the stress-strain curve was relatively straight before a sudden fall-off. By contrast, for in-phase or anti-symmetric unit cells, the shear matrix pocket cracking and interface shear failure propagating from the fill tow edge line crossing the warp tow that met the matrix pocket cracking were the main failure modes. Shear failure also started in the warp tows, owing to the large shear deformation of the fiber tows but developed gradually and was not fully completed before the interface separation failure. The stress-strain curve appeared less stiffness than that of the symmetric case and showed a large degree of nonlinearity because of the gradual development of shear failure. For the two-ply unit cell shifted by a quarter wavelength of the warp tow, the failure behavior was found to be between the above two cases. Tensile stress-dominated interface failure occurred in the region where the distance between the warp tows of adjacent plies was the smallest, followed by the gradual development of shear failure. The initial stiffness was the highest in the symmetric case, and peak stress was the highest in the in-phase case, whereas it was intermediate in the shifted case.

Internal pre-defects were found to affect the failure behavior. Of the considered defects, matrix pocket void and fiber-tow–matrix-pocket separation affected the failure progression and reduced the peak stresses significantly, depending on the fiber tow arrangement as well as the defect location. The largest effect occurred when the defect was placed not at the region where the interface failure started for the no-defect case, but in the region next to it, where the growth of the interface separation failure was resisted due to changes in the failure mode. One exception was the matrix pocket void placed at the crown part of warp tows (Region 1) for the symmetric case, in which a large amount of warp tows straightening due to the removal of the matrix in that region, together with contact between the fill tows of adjacent plies, accelerated the interface separation failure. Void fractions of 2.4–3% were found to reduce the peak stress by as much as 25.7%, 7.35%, and 18.4% for the symmetric, in-phase, and shifted cases, respectively.

## Figures and Tables

**Table 1:** Dimensions of fiber tows.

$\lambda_{w}=\lambda_{f}$ (mm)	1.55
$t_{w}=t_{f}$ (mm)	0.13

**Table 2:** Elastic properties.

Fiber tow (AS4/3501-6)	E1 (GPa)	156
	E2 = E3 (GPa)	10.4
	G12 = G13 (GPa)	5.9
	G23 (GPa)	2.84
	ν12 = ν13	0.256
	ν23	0.44
Matrix (3501-6)	E (GPa)	3.8
	ν	0.34

**Table 3:** Cohesive properties.

Material	Failure Mode	*T*_1_ (MPa)	*T*_2_ = *T*_3_ (MPa)	*G_IC_* (N/mm)	*G_IIC_* = *G_III_* (N/mm)
Warp/fill	Fiber failure Matrix failure	2310 60	- 90	30 0.1	- 1.0
Matrix	Matrix failure	60	110	0.2	1.0
Interface	Separation	60	90	0.1	1.0

**Table 4:** Peak nominal stresses and their percent reduction compared to no defect cases for symmetric and in-phase plain weave unit cells.

Defect Code	Symmetric		In-Phase	
	$\mathrm{Peak}\;\sigma_{xx}\;\;(\mathrm{MPa})$	Reduction (%)	$\mathrm{Peak}\;\sigma_{xx}\;\;(\mathrm{MPa})$	Reduction (%)
MPV1 MPV2 MPV3 MPV4	443.38 433.15 443.01 334.64	2.84 5.08 2.92 25.74	534.26 523.34 549.43 505.01	3.60 5.57 0.86 7.35
TMS1 TMS2 TMS3 TMS4	406.62 369.86 370.49 392.88	10.09 18.95 18.81 13.91	507.34 474.19 504.86 505.10	8.46 14.44 8.90 8.86
WFS1 WFS2 WFS3 WFS4	451.97 448.69 456.11 454.80	0.81 1.64 0.02 0.30	522.18 517.75 525.60 547.99	5.78 6.58 5.17 1.13
MCW1 MCW2 MCW3 MCW4	456.20 456.04 455.57 455.67	0.00 0.03 0.14 0.11	545.25 554.10 543.25 551.77	2.16 1.83 1.98 0.44
MCF1 MCF2 MCF3 MCF4	455.60 447.30 455.89 455.15	0.16 1.98 0.10 0.26	549.03 553.00 547.16 552.50	0.94 0.22 1.28 0.31

**Table 5:** Peak nominal stresses and their percent reduction compared to no defect cases for two-ply shifted plain weave unit cell.

Defect Code	$\mathrm{Peak}\;\sigma_{xx}\;\;(\mathrm{MPa})$	Reduction (%)
MPV21 MPV22 MPV23 MPV24 MPV25	481.30 482.28 424.78 419.04 468.85	6.24 6.04 17.25 18.36 8.66
TMS21 TMS22 TMS23 TMS24 TMS25	511.23 492.13 436.24 422.91 486.93	0.40 4.13 15.01 17.61 5.14

## Data Availability

The data presented in this study are available upon request from the corresponding author.

## References

[1] Miao Y., Zhou E., Wang Y., Cheeseman A. (2008). Mechanics of textile composites: Micro-geometry. Compos. Sci. Technol..

[2] Selver E., Potluri P., Hogg P., Soutis C. (2016). Impact damage tolerance of thermoset composites reinforced with hybrid commingled yarns. Compos. B.

[3] Priyanka P., Dixit A., Mali H.S. (2017). High-strength hybrid textile composites with carbon, kevlar, and e-glass fibers for impact-resistant structures. A review. Mech. Compos. Mater..

[4] Miravete A. (1999). 3-D Textile Reinforcements in Composite Materials.

[5] Liu Y., de Araujo M., Hu H. (2016). Advanced fibrous architectures for composites in aerospace engineering. Advanced Composites Materials for Aerospace Engineering: Processing, Properties and Application.

[6] Ishikawa T., Chou T.W. (1982). Elastic behavior of woven hybrid composites. J. Compos. Mater..

[7] Ishikawa T., Chou T.W. (1982). Stiffness and strength behavior of woven fabric composites. J. Mater. Sci..

[8] Tan P., Tong L., Steven G.P. (1997). Modelling for predicting the mechanical properties of textile composites—A review. Compos. A.

[9] Lomov S.V., Huysmans G., Luo Y., Parnas R.S., Prodromou A., Verpoest I., Phelan F.R. (2001). Textile composites: Modelling strategies. Compos. A.

[10] Karkkainen R.L., Sankar B.V. (2006). A direct micromechanical method for analysis of failure initiation of plain weave textile composites. Compos. Sci. Technol..

[11] Woo K., Whitcomb J.D. (1996). Three-dimensional failure analysis of plain weave textile composites using a global/local finite element method. J. Compos. Mater..

[12] Ernst G., Volger M., Huhne C., Rolfes R. (2010). Multiscale progressive failure analysis of textile composites. Compos. Sci. Technol..

[13] Woo K. (2017). Fracture analysis of woven textile composite using cohesive zone modeling. J. Mech. Sci. Technol..

[14] Choi K.H., Hwang Y.T., Kim H.J., Kim H.S. (2019). Progressive failure analysis of woven composites considering structural characteristics based on micro-mechanics. Compos. Struct..

[15] Cantwell W.J., Morton J. (1992). The significance of damage and defects and their detection in composite materials: A review. J. Strain. Anal. Eng. Des..

[16] Garnier C., Pastor M.-L., Eyma F., Lorrain B. (2011). The detection of aeronautical defects in situ on composite structures using non destructive testing. Compos. Struct..

[17] Lee D.H., Lee W.I., Kang M.K. (2006). Analysis and minimization of void formation during resin transfer modeling process. Compos. Sci. Technol..

[18] Mehdikhani M., Gorbathikh L., Verpoest I., Lomov S.V. (2019). Voids in fiber-reinforced polymer composites: A review on their formation, characteristics, and effects on mechanical performance. J. Compos. Mater..

[19] Krumm M., Sauerwein C., Hammerle V., Oster R., Diesel B., Sindel M. (2012). Capabilities and application of specialized computed tomography methods for the delamination of characteristic material properties of fiber composite components. Lock-In Thermography.

[20] Hart K.R., Chia P.C.L., Sheridan L.E., Wetzel E.C., Sottos N.R., White S.R. (2017). Mechanisms and characterization of impact damage in 2D and 3D woven fiber-reinforced composites. Compos. A.

[21] Nega B.F., Woo K., Lee H. (2019). Test and analysis of triaxially braided composite circular arch under three-point bending. Compos. Res..

[22] Catche S., Piquet R., Lachaud F., Castanie B., Benaben A. (2015). Analysis of hole wall defects of drilled carbon fiber reinforced polymer laminates. J. Compos. Mater..

[23] Liu L., Zhang B., Wang D., Wu Z. (2006). Effect of cure cycles on void content and mechanical properties of composite laminates. Compos. Struct..

[24] Landry B., Hubert P. (2015). Experimental study of defect formation during processing of randomly-oriented strand carbon/PEEK composites. Compos. A.

[25] Talreja R. (2013). Studies on the failure analysis of composite materials with manufacturing defects. Mech. Compos. Mater..

[26] Almeida S.F.M., Neto Z.S.N. (1994). Effect of void content on the strength of composite laminates. Compos. Struct..

[27] Senthil K., Arockiarajan A., Palaninathan R., Santhosh B., Usha K.J. (2013). Defects in composite structures: Its effects and prediction methods—A comprehesive review. Compos. Struct..

[28] Woo K., Nelson J.W., Cairns D.S., Riddle T.W. Effects of defects: Part B-Progressive damage modeling of fiberglass/epoxy composite structures with manufacturing induced flaws utilizing cohesive zone models. Proceedings of the 54th SDM Conference, AIAA 2013-1628.

[29] Cairns D.S., Nelson J.W., Woo K., Miller D. (2016). Progressive damage analysis and testing of composite laminates with fiber waves. Compos. A.

[30] Ashir M., Mocke A., Cherif C. (2019). Effect of the position of defined local defect on the mechanical performance of carbon-fiber-reinforced plastics. AUTEX Res. J..

[31] Hufenbach W., Bohm R., Gude M., Berthel M., Hornig A., Rucevskis S., Andrich M. (2012). A test device for damage characterization of composites based on in situ computed tomography. Compos. Sci. Technol..

[32] Dangora L.M., Mitchell C.J., Sherwood J.A. (2015). Predictive model for the detection of out-of-plane defects formed during textile-composite manufacture. Compos. A.

[33] Stamopoulos A.G., Ilio A.F. On the predictive tools for assessing the effect of manufacturing defects on the mechanical properties of composite materials. Proceedings of the 12th CIRP Conference on Intelligent Computation in Manufacturing Engineering.

[34] Ma Y., Li S., Wang J., Ju L., Liu X. (2018). Influence of defects on bending properties of 2D-T700/E44 composites prepared by improved compression molding process. Materials.

[35] Woo K., Peterson W.M., Cairns D.S. (2014). Selective activation of intrinsic cohesive elements. J. Appl. Math. Phys..

[36] Woo K., Cairns D.S. (2019). Selective activation of intrinsic cohesive elements for fracture analysis of laminated composites. Compos. Struct..

[37] Tang X., Whitcomb J.D. (2003). Progressive failure behaviors of 2D woven composites. J. Compos. Mater..

[38] Mollon V., Vina J., Arguelles A., Bonhomme J., Vina I. (2011). Influence of the mode mixity ratio and test procedures on the total energy release rate in cabon-epoxy laminates. Proc. Eng..

[39] Xia Z., Zhou C., Yong Q., Wang X. (2006). On selection of repeated unit cell model and application of unified periodic boundary conditions in micro-mechanical analysis of composites. Int. J. Solids Struct..

[40] Jacques A., Baere I.B., Paepegem W.V. (2014). Application of periodic boundary conditions on multiple part finite element meshes for the meso-scale homogenization of textile fabric composites. Compos. Sci. Technol..

[41] (2014). Abaqus 6.14 Documentation.

[42] Turon A., Davila C.G., Camanho P.P., Costa J. (2007). An engineering solution for mesh size effects in the simulation of delamination using cohesive zone models. Eng. Fract. Mech..

[43] Pandolfi A., Ortiz M. (1998). Solid modeling aspects of three-dimensional fragmentation. Eng. Comput..

[44] Paulino G.H., Celes W., Espinha R., Zhang Z. (2008). A general topology-based framework of adaptive insertion of cohesive elements in finite element meshes. Eng. Comput..

[45] Hashin Z. (1980). Failure criteria for unidirectional fibre composites. J. Appl. Mech..

